# Academic Pediatric Surgery Capacity Building in Vietnam Through PASS, a Pediatric Acute Surgical Support Course

**DOI:** 10.3389/fsurg.2022.868483

**Published:** 2022-04-20

**Authors:** Bich-Uyen Nguyen, Aixuan Holterman, Mark Holterman, Le-Thanh Dinh

**Affiliations:** ^1^ Department of Pediatric Surgery, Ho Chi Minh University of Medicine and Pharmacy, Ho Chi Minh City, Vietnam; ^2^ Department of Surgery at Peoria and Chicago; Department of Pediatrics at Chicago, University of Illinois College of Medicine at Peoria and Chicago, Chicago, IL, United States; ^3^ Department of Pediatric Surgery, Children’s Hospital 1, Ho Chi Minh City, Vietnam

**Keywords:** Vietnam 1, pediatric surgical emergencies 2, Training 3, capacity building 5, pediatric emergencies 6, global health

## Abstract

Neonatal and pediatric surgical emergencies in Low and Low Middle Income countries remain a significant challenge in combatting the burden and inequities of global health. IPSAC-Vietnam is a small Non-Governmental Organization that has been engaged in a 12-year multi-pronged partnership with several children’s hospitals in Vietnam VN to enhance pediatric surgery capacity. We describe the health care, medical training and emergency system in VN as the background for IPSAC activities and development of Pediatric Acute Surgical Support (PASS) course. The course goal is to prepare health care personnel in the immediate management of neonatal/pediatric life-threatening surgical conditions and road injuries at their first point of entry into Vietnam hospitals. PASS is a horizontal outreach initiative that adopts an interprofessional, multidisciplinary, team-training, train-the-trainers, and outcome-based training approach. PASS can be used as a tool for sustainable horizontal capacity-building by champion leaders at the teaching children’s hospitals and medical universities in developing countries, to strengthen training for pediatric surgical emergencies, to integrate pediatric and pediatric surgical care and to advocate for a comprehensive approach to emergency care of the critically ill child.

## Background

We first explain the context in which IPSAC-VN (International Specialist Alliance for the Children of Vietnam, www.ipsacvietnam.org) Non- Governmental Organization (NGO) functions by describing the health care and training system in Vietnam (VN) to better understand VN pediatric surgery and emergency care.

### Health care System in VN

At the heel of its adoption of a market-driven economy, VN gross national income per capita for a population of ∼98.5 million citizens grew from 410 USD in 2000 to 2,660 USD in 2020 (https://data.worldbank.org/indicator/NY.GNP.PCAP.CD?locations = VN). Consequently, the World Bank’s previous designation of VN as a Low-Income-Country has changed to that of a Low-Middle-Income-Country LMIC. As the socioeconomic conditions improve, VN experiences the same worldwide shift from communicable to non-communicable pathologies, with more recognition for the burden of unmet surgical needs (1), and emergency conditions in the acutely ill and injured child (2–5).

We focus our description on the South of VN where our NGO activities predominate, but surmise that the medical system in the remaining VN shares similar characteristics. Children in urban Ho Chi Minh City HCMC (1) (9.1 million inhabitants) as well as the remaining South and Central VN are served at the central children’s hospitals (CH), the two 1,500-beds CH1 and CH2, and the new 1,000-beds City Children’s Hospital (CCH). These CHs provide tertiary care for children under 15 years and offer graduate and postgraduate training for the HCMC University of Medicine and Pharmacy (UMP), the Pham Ngoc Thach Medical University (PNT) and the Faculty of Medicine at the University of Science. In 2007 when IPSAC first met with CH1, CH1 was the referral center for 31 provinces, with a daily outpatient visit of 3,500–5,200 children, a yearly admission of 65,000 patients and surgical volume of 20,000 cases. The lower-level facilities are the provincial pediatric hospitals (population of >1.5 million), the adult district hospitals (population of about 150,000) and the community hospitals.

Under the VN health insurance policy, only children under six get free medical care (6). Older children receive government supplements but primarily pay out-of-pocket for health care. Private health insurance is available for the affluent, but is mostly limited to outpatient elective care (7). Pediatric health care is under the direction of the Ministry of Health MOH but is decentralized. The provincial and district government have the authority to manage their health facilities and training activities. The local medical providers have open-ended fee-for-service reimbursement for hospital-based care. These factors introduce potential financial disincentives for out-of-hospital referral from the lower-level hospitals, as well as motivation for tertiary level hospitals to maintain a high patient volume (7, 8).

The designated first stop for pediatric patients from rural VN is the local district or community hospital. As with most LMIC (9), VN is challenged with disparities in workforce skills and limited availability of pediatric surgery and pediatric disciplines that adversely affect the safety and quality of pediatric surgical care. The primary and secondary hospitals control the referral process to the next tiered facilities, but not patient flow. Families from rural or provincial VN have limited confidence in and often bypass their regional facilities to self-refer to the tertiary hospitals, at the expense of extensive travel, delayed clinical evaluation and definitive treatment, loss of income and support network, and the added costs of user fees for bypassing the referral system. This lack of trust in the local hospitals further strains the overloaded capacity at the tertiary CHs. These socioeconomic factors contribute to worse health outcome for the acutely ill poor from remote VN (10, 11).

Pediatric emergency and critical care are the most vulnerable points of the patients’ first point of entry into the health care system, particularly for road injury which is the leading cause of pediatric mortality (12–15). In response to these imbalances, the MOH established the Direction of Health Care Activities (DOHA) policy, tasking high-level hospitals for clinical support, skill and technology transfer to their affiliated hospital network (16).

### Medical Education and Postgraduate Training in VN

Medical training is offered at 29 medical universities for a 4-years or a 6-years program. The curriculum is not standardized and without national licensing process (17). Graduates from the 4-year MD program mostly come from remote VN. This 4-year program is being phased out at most universities, to extend the training into a new 6-year program for students from the provinces. These students have a service contract with their local hospitals in exchange for medical school expense support. The medical school classrooms are overcrowded with class size of 400–800 students and a low faculty-student ratio. Students are taught through the traditional didactic lectures (18). Medical universities have recently been pursuing curriculum reform aiming for student-oriented competency-, evidence- and problem-based teaching (19).

The postgraduate medical training system is complex, unregulated, and without licensing or national examination. Most of the graduates (90%) seek medical residencies in various hospitals for a medical diploma that clears them to practice medicine after 18 months. They can next enroll for Level 1 Specialist after another 24 months, and Level 2 Specialist with an additional 2 years of training. The remaining paths are the academic track leading to a 2-year Master degree and a 3–5 year PhD degree; or the competitive 3-year “Resident” Physician Specialist level 1 track with the option of a Master and PhD degree (17).

The VN government recently decreed that Continuing Medical Education CME certification is required for license maintenance, and assigned the responsibilities of developing and providing CME to leading medical institutions (20). The CME infrastructure is however still underdeveloped, unstandardized with the same learning approach of lecture hall didactic teaching.

### Pediatric Surgery and Emergency Medicine in Vietnam

The HCMC UMP is the only program in VN with a formal pediatric surgery training curriculum and the ability to award an official pediatric surgery diploma. It graduates 5–6 pediatric surgeons/year. The overwhelming majority of the pediatric surgical trainees apply for a surgical apprenticeship at various children’s hospitals where directors of pediatric surgery have independent specialist degree-conferring capacity. The Pediatric Surgery departments generally do not have an organized curriculum, although UMP faculty have shared their teaching activities with hospital trainees.

Graduates from large tertiary CHs generally have stronger technical skills. Because of the complexity of the training system, the skill and knowledge levels among the graduates are uneven, and the numbers of “pediatric surgeons” are inexact, although the broad estimate for Central and South VN surgeons with any pediatric surgical training level is 230 surgeons. The training is inbred and authoritative. It is heavily focused on surgical techniques and less on clinical decision-making and patient management skills. Children with surgical problems in major cities will most likely be treated by well-trained and experienced pediatric surgeons. At lower-level facilities in the provinces, especially in remote areas, children will be cared for by general surgeons.

As previously mentioned, the burden of pediatric emergency care and injuries in VN is high. Road traffic injuries are expected to rise as the use of motor vehicles increases. The ambulance service has protracted personnel and equipment shortages and is impeded by severe urban traffic congestion. Fewer than 4% of road injuries use ambulance transport, and less than 40% of the trauma victims receive on-the-scene care by the Emergency Medical Service (14). There is no dedicated prehospital care, coordination of clinical services or trauma system for pediatric patients. Injured children are more often brought by the family’s motorcycle, or by taxis in the “scoop and run” manner to the closest hospital (15). Trauma theory is taught with didactic lectures that are focused on individual organ injuries, and not on a systematic approach to life-threatening injuries and teamwork. Emergency medicine is not appreciated as a specialty until after 2010, with the first residency program established in 2013 (21). A training program in Critical Care-Emergency Medicine-Poison Control is being developed at UMP. The American Heart Association BLS and ACLS provider courses are offered along with instructor training in emergency programs, but APLS and PALS training have yet to be consistently organized, with efforts mostly provided ad hoc by international and local philanthropic organizations.

In summary, the quality of pediatric care is inequitable, with the highest burden for the poor living away from the central children’s hospitals. The tertiary children’s hospitals are overwhelmed with patient volume and struggle to keep up with the service demands. There are needs for integrated and patient-centric pediatric surgical and emergency care. Graduate and postgraduate training are uneven and can benefit from learners’-oriented training for critical thinking, problem-solving, self-learning and collaborative care. These characteristics serve as the background for the focus of IPSAC-Vietnam volunteer efforts.

## IPSAC-VN’s Global Health Activities

IPSAC-VN is a small 501c3 NGO that was established in 2009 with the mission to enhance the care of the children in VN through partnership with Vietnamese professionals to strengthen the workforce and institutional capacity. Its activities are predominantly in South VN where IPSAC-VN initiated the collaboration. IPSAC-VN international multidisciplinary visiting team volunteered time, effort and expenses yearly or biyearly for a week’s time. It is normally comprised of pediatric nurses, pediatric surgical and pediatric specialists (anesthesia, neonatology, hematology-oncology, etc.), most of whom hold academic positions at medical institutions. IPSAC first Memorandum of Understanding with CH1 was focused on education, training and academic support for the CH1 pediatric surgery section in HCMC, in alignment with IPSAC-VN academic strengths. We next describe the evolution of IPSAC-VN efforts.

### Phase 1 of IPSAC-VN Activities

The initial capacity building engagement were mission-based and vertical in nature, with regular clinical interactions, exchanging specialized surgical and patient management skills to complement the surgical care at CH1. IPSAC also provides mentorship and academic support for nurses and surgeons by sponsoring international clinical rotations and professional meetings to enhance their out-of-the country perspectives, and supporting their academic output in peer-reviewed journals (22–27). Over the first 3–5 years of on-the-ground efforts, our personal relationship with the VN colleagues solidified; and our understanding of the local health care system, of the hospital infrastructure and of the needs of our VN partners evolved.

### Phase 2 of IPSAC-VN Activities

**Figure 1:**
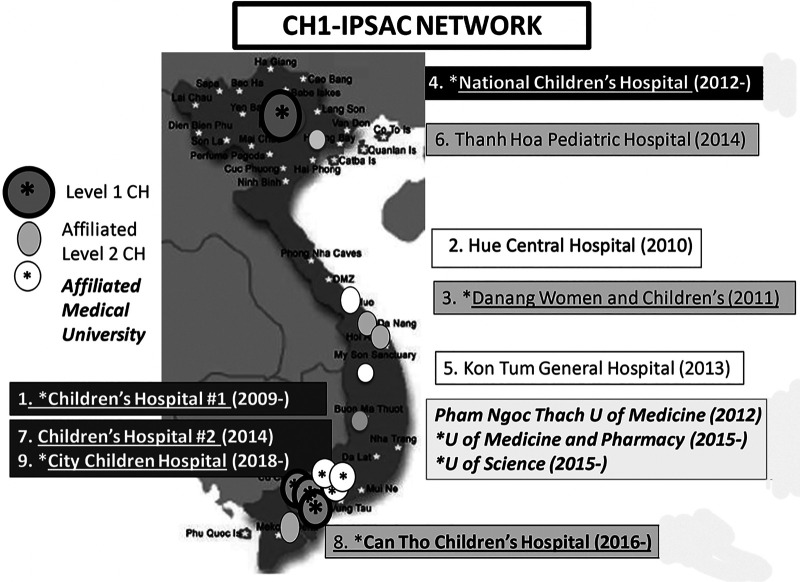
Map of Vietnam and sites of IPSAC-VN activities in Vietnam. Level 1 Children's Hospital CH (in shading circle and asterisk in the map); the National Children's Hospital 1 NCH in the capital city of Hanoi, and Children's Hospital 1 (CH1), Children's Hospital 2 (CH2), City Children's Hospital. (CCH) in Ho Chi Minh City. Level 2 CH (in shading ellipse in the map); with Danang Women and Children's Hospital DWCH, Thanh Hoa Pediatric Hospital and Can Tho Children's Hospital (CTCH). The adult hospitals (in white circle in the map); with Hue Central Hospital and Kon Tum General Hospital. The teaching universities (in white circle and asterisk in the map); Pham Ngoc Thach University of Medicine, University of Medicine and Pharmacy; University of Science. The initiating years of the activities are depicted, with ongoing efforts at 1. CH1, 3. DWCH, 4. NCH, 8. CTCH, 9. CCH.

To alleviate heavy patient flow from the provinces and to reduce CH1 clinical capacity exhaustion, CH1 and IPSAC joint capacity building activities were expanded during the next phase of collaboration to provincial pediatric hospitals affiliated with CH1, primarily in the central coast of Vietnam (Danang City), the highlands (Kon Tum) and the deep South (Can Tho City) (Figure 1 for IPSAC sites). The visiting team is normally composed at the minimum of CH1 and IPSAC pediatric surgeons, pediatric anesthesiologists, and IPSAC pediatric nurses and neonatologists. While multidisciplinary patient management is emphasized, the technical training focus is site-dependent, ranging from open to laparoscopic neonatal and pediatric surgical index cases.

**Figure 2:**
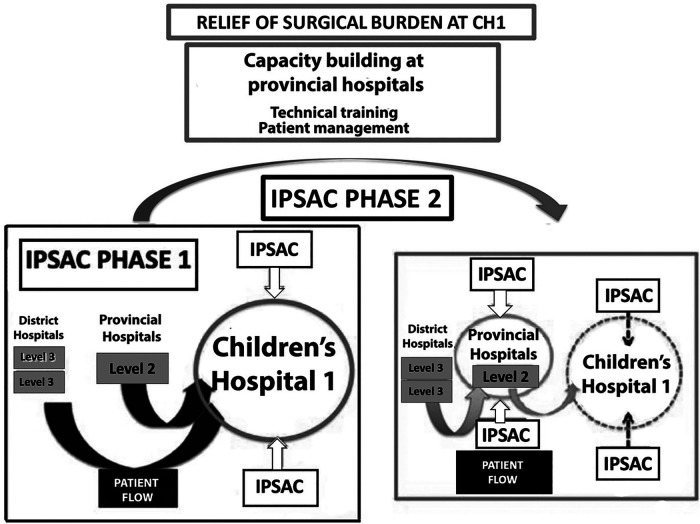
Diagram of Phases 1 and 2 of CH1-IPSAC joint surgical capacity building at the affiliated hospitals to relieve patients’ surgical burden.

This clinical expansion met the MOH DOHA decree that CH1 increases clinical support for its regional affiliates. As a result of these efforts, coupled with organized public information campaigns by the provincial hospital leadership of the team schedule, the local surgical capacity improved for the appropriate pathologies, limiting the families’ travel burden, and decreasing patient flow and workload at CH1 (Figure 2).

### Current Phase of IPSAC Activities

In the last 5 years, we extended our building capacity activities to basic science research (23) and applied research such as a multi-center randomized clinical trial for Biliary Atresia at the National CH in Hanoi and CH1, using a repurposed medication (28). We assisted the pediatric surgical faculty at UMP in reforming pediatric surgical training to standardize and update its curriculum and define training goals for HCMC pediatric surgeons. We conducted regular teleconferences for mentoring and case-based learning to enhance clinical decision-making. Some of the measurable impact of the academic efforts include engaging pediatric surgeons in cross-disciplinary research collaborations (hepatology, critical care, hematology, radiology, and pathology support for the clinical trial) and clinical trial funding for the local principal investigators (28); private industry support for translational basic science projects at the University of Science, and Vietnam Education Foundation Grant from the US Government for the pilot project in the PASS Pediatric Acute Surgical Support course.

## PASS-Pediatric Acute Surgical Support

PASS is an interprofessional training course in pediatric surgical emergencies to address inefficiencies in VN hospital pediatric surgical emergency care and team approach in coordinating the care of the surgical child. One of the goals is to reduce preventable pediatric surgical mortality and morbidity, by preparing health care providers in the initial assessment, resuscitation and stabilization of children with life-threatening general surgical illnesses. PASS is an evidence-based, context-appropriate course with blended teaching approaches. The course contents include lecture materials about the fundamentals of neonatal/pediatric surgical emergencies that can be delivered remotely as didactic presentations; on-site standardized interactive practical skill stations, clinical scenario discussions, unfolding trauma scenarios, and learners’ and course performance evaluations. The core lectures consist of principles of trauma resuscitation and organ system management, and emergency surgical pathologies of the neonate, infant, and child. PASS was developed at PNT in 2016–2017 and implemented at CH1 and CCH between 2017–2019. Of note, PASS is not a surgical procedural skill courses or first-aid preparation in pre-hospital care. PASS is not duplicating the World Health Organization LMIC emergency care and trauma training since it is pediatric surgery-centric. PASS has the following distinguishing features:

i. Collaborative design. The course was developed by a multiinstitutional team of CH1 pediatric surgeons and critical care specialists along with US collaborators from different disciplines.
ii. LMIC-centric and context-appropriate.
  A. CH1 instructors are critical to the course design for identifying clinical gaps and the best way to deliver the course.
  B. PASS applies cost saving measures such as didactic lecture review by VN instructors before the international team arrival; use of existing spaces and teaching tools without the need for SIM facilities, on-the-ground personnel for logistic support.
  C. PASS has flexibility in the course schedule and contents to meet the needs of the local institutions.
  D. The teaching materials are bilingual and language translators are used for the on-site course.
iii. Practical learning.
  A. The emphasis is on the critical assessment and management nodes beyond the core lectures of theoretical aspects of pediatric surgery.
  B. The on-site teaching activities are focused on problem-solving, role-playing exercises during team-based simulated trauma case scenarios to promote critical thinking.
  C. PASS emphasizes the systematic approach to life-threatening injuries/illnesses assessment and treatment.
iv. Interactive. PASS promotes learner-teacher interactions.
  A. by bidirectional clinical case scenario discussions.
  B. by immediate team performance feedback and debriefing of simulated trauma scenarios.
v. Fostering the spirit of team work (29).
  A. PASS engages physicians and nurses in team building exercises and team communication.
  B. PASS empowers nurses in team clinical decision making to promote the importance of physician extenders in good patient outcome.
vi. Outcome-based.
  A. PASS incorporates short-term performance measures of didactic knowledge, *a priori* evaluation check list for patient management performance and team dynamics, and satisfaction surveys of the course experience.
  B. PASS encourages learners to identify gaps that can lend themselves to quality improvement and fundable research projects.
vii. Train-the-trainer approach.
  A. PASS was deployed at the high-level sites to recruit future local PASS advocates: at teaching universities to promote PASS incorporation into the postgraduate medical school curriculum, and at hospitals to expand PASS to their regional affiliates.
  B. PASS freely shares the teaching materials with the future local instructors.
viii. Impact. The immediate impact is professional development for front-line health care providers and institutions, including a new appreciation for interdisciplinary collaboration and teamwork that is not a traditional aspect of clinical care at teaching institutions in VN.
ix. A scalable project to sustain PASS impact. At the training level, the PASS course can be expanded into secondary and primary hospitals within VN and beyond. At the research level, secondary outcome research projects, and best practice protocols, databases and monitoring tools with measurable performance metrics and patient outcome indicators such as transfer mortality morbidities can be designed. Such data can be used as springboards to grant applications, larger scale partnership and/or to advocating for pediatric health policies.
x. PASS can be used as a tool for horizontal capacity building, since quality trauma and emergency care are an integral part of functional health systems.

Because of the last 2 years disruption in international travel, learners’ course retention could not be assessed. VN instructors have however continued to use PASS didactic materials and case discussion scenarios for providers at their own institutions.

## Limitations

IPSAC’s initial agenda was narrow with a vertical “mission”-based undertaking. As IPSAC-VN matured over the years, it however quickly adapted to horizontal capacity-building activities in program development, showing the need for adaptability and agility of a small NGO to evolve to better maximize its impact.

As an NGO with strong representation of academic pediatric specialists, IPSAC-VN uses within its means a targeted academic approach to education, training and research to achieve the goals of enhancing the workforce and LMIC institutional capacity. Since the efforts are outside organized official institutional support, IPSAC-VN lacks the robust assets for structured longitudinal partnerships at the institutional level. This level of partnership can better support robust outcome research initiatives to demonstrate the health impact of the joint outreach program-building activities. Objective outcome measures such as for example, enhanced surgical volume and perioperative morbidities/mortality for specific pediatric surgical conditions; and as mentioned for the PASS educational intervention, changes in patient transfer mortality or the learners’ practice behavior in pediatric trauma care can be incorporated into our program evaluation.

PASS feasibility and short-term preliminary efficacy analyses are restricted to learners’ immediate course performance and course satisfaction from Vietnam high level pediatric hospitals (30). To further evaluate its applicability and acceptability outside VN, we anticipate offering PASS to other LIMC institutions in Africa and South Asia, with additional plans to incorporate outcome assessment tools at the patient care level to the program.

## Concluding Remarks. Lessons Learned and Aspired Goals

Based on our experience, the critical determinants for a lasting partnership are: (1) building and maintaining strong personal relationship with the local partners; (2) identifying stakeholders who have shared goals, and who serve as local champions to take ownership of the program(s); (3) adopting strategic joint programs that are relevant to our partners’ needs; (4) judicious use of the best resources (in IPSAC-VN case, mostly skilled clinicians and academicians); (5) selecting projects with feasible scope and achievable measurable impact, or of projects that can be scaled up such as PASS.

IPSAC-VN recommends and encourages participation of the academic surgical community, of academic institutions and larger organizations in LMIC program building efforts. They can best mobilize and apply their vast resources to better address the complexity of engagement at the level of LMIC health systems to maximize the creation of sustainable global health programs (31, 32).

As IPSAC-VN list of international volunteer partners grows, it has the opportunity through volunteers from academic pediatric surgical institutions to initiate structured twinning programs to broaden IPSAC-VN services. For the next decade, IPSAC will continue to promote the culture of comprehensive patient care and teamwork, as well as of an academic approach to patient care, pediatric surgery training, program development and health outcome research.

## Data Availability

The original contributions presented in the study are included in the article/supplementary material, further inquiries can be directed to the corresponding author/s.
